# Determining essential dimensions for the clinical approximation of personality disorder severity: multi-method study

**DOI:** 10.1192/bjp.2025.10347

**Published:** 2026-01

**Authors:** André Kerber, Caroline Macina, Ludwig Ohse, Leonie Kampe, Oliver Busch, Michael Rentrop, Christine Knaevelsrud, Johannes Wrege, Susanne Hörz-Sagstetter

**Affiliations:** Division of Clinical Psychological Intervention, Freie Universität Berlin, Berlin, Germany; German Center for Mental Health (DZPG), Partner Site, Berlin–Potsdam, Berlin, Germany; University Psychiatric Clinics (UPK) Basel, Basel, Switzerland; Department of Clinical Psychology and Psychotherapy, Psychologische Hochschule Berlin, Berlin, Germany; Department of Psychology, MSB Medical School Berlin, Berlin, Germany; Department of Psychiatry, Psychotherapy, and Psychosomatic Medicine, Klinikum Itzehoe, Itzehoe, Germany; Department of Psychiatry, Hospital Emmental, Langnau, Switzerland; KBO-Inn-Salzach Klinikum, Wasserburg am Inn, Germany; Department of Psychiatry and Psychotherapy, Technical University of Munich, Munich, Germany

**Keywords:** Personality disorder severity (PDS), DSM-5 alternative model for personality disorders (AMPD), criterion A, dimensional assessment of personality disorder, personality disorder in ICD-11

## Abstract

**Background:**

Decades of research on the dimensional nature of personality disorder have led to the replacement of categorical personality disorder diagnoses by a dimensional assessment of personality disorder severity (PDS) in ICD-11, which essentially corresponds to personality functioning in the alternative DSM-5 model for personality disorders. Besides advancing the focus in the diagnosis of PD on impairments in self- and interpersonal functioning, this shift also urges clinicians and researchers worldwide to get familiar with new diagnostic approaches.

**Aims:**

This study investigated which PDS dimensions among different assessment methods and conceptualisations have the most predictive value for overall PDS.

**Method:**

Using semi-structured interviews and self-reports of personality functioning, personality organisation and personality structure in clinical samples of different settings in Switzerland and Germany (*n* = 534), we calculated a latent general factor for PDS (g-PDS) by applying a correlated trait correlated (method – 1) model (CTC(M–1)).

**Results:**

Our results showed that four interview-assessed PDS dimensions: defence mechanisms, desire and capacity for closeness, sense of self, and comprehension and appreciation of others’ experiences and motivations account for 91.1% of variance of g-PDS, with a combination of either two of these four dimensions already explaining between 81.8 and 91.3%. Regarding self-reports, the dimensions depth and duration of connections, self-perception, object perception and attachment capacity to internal objects predicted 61.3% of the variance of a latent interview-based score, with all investigated self-reported dimensions together adding up to 65.2% variance explanation.

**Conclusions:**

Taken together, our data suggest that focusing on specific dimensions, such as intimacy and identity, in time-limited settings might be viable in determining PDS efficiently.

The implementation of a dimensional assessment of personality disorder severity (PDS) in the ICD-11^1^ and the Alternative Model for Personality Disorders (AMPD) in the DSM-5^2^ represents a crucial step towards an empirically based model for the diagnosis of personality disorder. This paradigm shift was motivated by the limitations of the categorical approach, such as high comorbidity and low specificity.^3,4^ According to the ICD-11, PDS is characterised by impairments in the functioning of the self (e.g. identity, self-worth, capacity for self-direction) and/or problems in interpersonal functioning (e.g. developing and maintaining close and mutually satisfying relationships, understanding others’ perspectives, managing conflict in relationships). This definition largely corresponds to personality functioning (see Table 1), as operationalised in the Level of Personality Functioning Scale (LPFS^5^) in the AMPD (for a detailed differentiation of the AMPD and personality disorders in ICD-11, see^6^). According to Criterion A of the AMPD, a personality disorder diagnosis requires at least moderate impairment in personality functioning. Using personality functioning as an indicator for PDS provides relevant clinical insights for individual treatment planning,^7^ high clinical utility^8^ and information on subthreshold personality difficulties.^9^

**Table 1:** Dimensions of personality disorder severity according to personality functioning,^2^ personality organisation^10^ and personality structure^11^ investigated in this study (marked in bold)

Personality functioning	Personality organisation	Personality structure
Identity **Sense of self** **Stability of self-esteem** **Emotional range and regulation** Self-direction **Ability to pursue meaningful goals** **Constructive, prosocial internal standards** **Self-reflective functioning** Empathy **Comprehension and appreciation of others’ experiences and motivations** **Tolerance of differing perspectives** **Understanding of effects of own behaviour on others** Intimacy **Depth and duration of connections** **Desire and capacity for closeness** **Mutuality of regard reflected in interpersonal behaviour**	**Identity** Capacity to invest Sense of self Representation of others **Object relations** Interpersonal relationships Intimate relationships and sexuality Internal investments in others **Defence mechanisms** Lower-level defences Higher-level defences **Aggression** Self-directed aggression Other-directed aggression **Moral values** **Narcissism**	**Self-perception** Self-reflection Affect differentiation Identity **Object perception** Self-object-differentiation Whole object perception Realistic object-perception **Self-regulation** Impulse control Affect tolerance Regulation of self esteem **Regulation of object relations** Protecting relationships Balancing of interests Anticipation **Internal communication** Experiencing affects Use of fantasies Bodily self **Communication with others** Making contact Communication of affect Empathy **Attachment capacity to internal objects** Internalisation Use of introjects (to calm or care for oneself) Variable attachments **Attachment capacity to external objects** Ability to make attachments Accepting help Severing attachments

## Measures for PDS and related constructs

The inclusion of a dimensional conceptualisation of personality disorder in the AMPD has led to the development of numerous new instruments to operationalise personality functioning, including validated self-report questionnaires: e.g. the LPFS-Self Report (LPFS-SR^12^) – and interviews: e.g., the Structured Clinical Interview for the Alternative DSM-5 Model for Personality Disorders – Module I (SCID-5-AMPD-I^13^), and the Semi-structured Interview for Personality Functioning DSM-5 (STiP-5.1^14^). Based on these measures, research on the validity and reliability of personality functioning as an indicator for PDS has been accumulated.^15–18^ The conceptualisation of personality functioning in DSM-5 is based on long-standing psychodynamic theories of personality pathology, including Kernberg’s model of personality organisation (see Table 1).^5,19^ Consequently, validated measures based on personality organisation, such as the Inventory of Personality Organization (IPO-30^20^) and the Structured Interview for Personality Organization – Revised (STIPO-R^10^), or related constructs, such as personality structure according to the Operationalized Psychodynamic Diagnosis (OPD) system^11^ with its OPD-Structure Questionnaire (OPD-SQ^21^), have shown high utility and convergence with personality functioning.^22^

## Determining the essential dimensions for the clinical approximation of PDS

The diversity of constructs and assessment methods for PDS necessitates identifying the dimensions that best capture its underlying latent construct. Previous research shows that PDS dimensions are highly correlated, reflecting a robust latent g-PDS.^15^ Identifying the empirically sound core dimensions can refine the theoretical understanding and can help improve assessment approaches, predictive validity and clinical utility in time-constrained settings. Although self-report questionnaires provide a practical means to approximate PDS, personality disorder is considered to be most reliably assessed by semi-structured interviews.^23,24^ Thus, it is important to determine which self-reported dimensions most closely align with interview-based assessments, thereby enhancing clinical efficiency and guiding future research. Based on validated assessments of PDS, this study therefore aimed to identify (a) the most central PDS dimensions for approximating g-PDS and (b) the self-reported dimensions that best approximate the interview-based g-PDS. To these aims, we combined data from two studies assessing a total of *n* = 534 participants with interviews (SCID-5-AMPD-I, STiP-5.1 and STIPO-R) and self-reports (LPFS-SR, IPO-30 and OPD-SQ) indicating PDS.

## Method

### Procedure and participants

Between May 2020 and June 2022, two multi-method studies were conducted in Germany and Switzerland with the combined data comprising *n* = 534 participants. The German clinical sample (*n* = 359) consisted of a mixed clinical out-patient and in-patient sample (for more information on recruitment process, see^25,26^). The Swiss sample consisted of out-patients from a psychiatric department of the University Clinics Basel (*n* = 177), including a non-clinical sample recruited via the University of Basel website (*n* = 29; for more information, see^27^). Overall, subjects were aged between 18 and 72.6 years (mean 32.6, s.d. = 11.7), thereof 66.9% women and 74.4% without children; 67.6% had regular work and 28.1% had a university degree. Overall, 57.7% of all available LPFS interviews scored above a global LPFS score of 1.5, indicating the presence of a personality disorder according to Buer Christensen et al.^28^ Overall, 43.6% were using psychotropic medication. The authors assert that all procedures contributing to this work comply with the ethical standards of the relevant national and institutional committees on human experimentation and with the Helsinki Declaration of 1975, as revised in 2008. All procedures involving human subjects were approved by the Ethics Committee of the Psychologische Hochschule Berlin (Nr. 2020-0214) and the Northwestern and Central Swiss Ethics Committee (Nr. 2020-02547). Written informed consent was obtained from all participants.

### Measures

#### STiP-5.1^14^

The STiP-5.1 is a semi-structured interview for the 12 subdomains and the total score of the LPFS, as defined in the DSM-5 AMPD (see Table 1). Each subdomain is rated from *no impairment* (0) to *extreme impairment* (4).^4^ We used the STiP-5.1 in the Swiss sample; for details on the assessment procedure, see Macina et al.^27^ The German version of the STiP-5.1 has excellent interrater reliability and good convergent validity.^29^

#### SCID-5-AMPD-I^13^

The SCID-5-AMPD-I is a semi-structured interview for the 12 subdomains of the LPFS (rated from *no impairment* (0) to *extreme impairment* (4)), as defined in the DSM-5 AMPD. The SCID-5-AMPD-I was used in the German sample, in which it showed excellent interrater reliability, mostly excellent test–retest reliability and good structural, convergent and discriminant validity (the detailed results have been published elsewhere^25^).

#### STIPO-R^10^

The STIPO-R is a semi-structured interview assessing personality organisation based on Kernberg’s object relations model.^30^ The STIPO-R contains 55 items, which can be rated on a 3-point scale, from *pathology absent* (0) to *significant to severe pathology* (2), and aggregated to six domains: identity, object relations, defence mechanisms, aggression, moral values and narcissism. In addition to the item scores, the interviewers provide a global clinical rating for each domain using anchor items scored from 1 (*no pathology*) to 5 (*very severe pathology*), similar to the procedure used in the above described LPFS interviews. For our analyses, we combined the global clinical ratings with the item-based domain scores through z-standardisation. The current sample comes from the validation study of the German STIPO-R, which demonstrated good to excellent interrater reliability, ranging from 0.57 for representation of others to 0.93 for self-directed aggression and shows strong convergence with other personality disorder measures.^26^ Further details can be obtained from the last author, S.H.-S., via the first author, A.K.

#### LPFS-SR^12^

The LPFS-SR is a self-report questionnaire capturing the 12 subdomains of the LPFS with high internal consistency and convergent validity. It includes 80 statements, which are rated on a 4-point Likert scale ranging from *totally false* (1) to *very true* (4). The German version of the LPFS-SR shows equally good internal consistency and convergent validity.^31^

#### OPD-SQ^21^

The OPD-SQ is a self-report assessment for personality structure according to OPD.^32^ The OPD-SQ comprises eight domains (see Table 1) with 95 items on a 5-point Likert scale ranging from *not true at all* (0) to *fully true* (4). Its scores show satisfying psychometric properties.^20^

#### IPO-30^20^

The IPO-30 measures the personality organisation domains of identity, primitive defences, aggression, moral values and reality testing, according to Kernberg’s object relations model.^29^ Participants rate 30 items on a 5-point Likert scale ranging from *never true* (1) to *always true* (5). The German version shows good psychometric properties.^20^

The German clinical sample completed two interviews (SCID-5-AMPD-I and STIPO-R) and three self-reports (OPD-SQ, IPO-30 and LPFS-SR) indicating PDS, while the Basel mixed sample completed one interview (STiP-5.1) and two self-reports (OPD-SQ and IPO-30) indicating PDS. Within the total sample of *n* = 534, the number of available assessments were *n* = 291 (LPFS interview: SCID-5-AMPD-I or STiP-5.1), *n* = 334 (LPFS-SR), *n* = 295 (STIPO-R), *n* = 465 (IPO-30) and *n* = 186 (OPD-SQ). The number of pairwise available assessments can be found in Supplementary Table 1 available at https://doi.org/10.1192/bjp.2025.10347. To address missing data, we employed a full information maximum likelihood (FIML) estimation in the following analyses, including robust standard errors to account for non-normal distributions of indicators. The fraction of missing information (FMI^33^), a measure of uncertainty introduced by missing data, is provided for all PDS dimensions in Supplementary Table 2. The total average FMI for all assessed PDS dimensions was 0.41, ranging from 0.22 (IPO-30 identity) to 0.56 (OPD-SQ internal emotional communication). According to Savalei & Rhemtulla^32^ the effective sample size was therefore equal to the dataset of *n* = 315 complete, non-missing cases (534 × (1 − 0.41)) which has enough power for estimating below described latent models.^34^ To further control for estimation uncertainty due to missing data, we corrected all reported confidence intervals for the width inflation factor (WIF; 1/sqrt(1-FMI)^33^) of the respective reported PDS dimension. A script containing the syntax for all following analyses is available in the Supplementary material. The data that support the findings of this study are openly available in the Open Science Framework at https://osf.io/bhq94/.

### Statistical analysis

Statistical analyses were conducted using the software R (2024; https://www.r-project.org)^35^ on Ubuntu Linux 22.04. We investigated the comparability of the samples with regard to the distribution of PDS among participants. The PDS scores were checked for normality utilising histograms and cut-offs for skewness <2 and kurtosis <7.^36^

### Identification of self- and interview-rating assessment method factors

To determine whether the assessment method introduces systematic variance, we first conducted a parallel analysis using five different total scores: an interview-based personality functioning score (derived from STiP-5.1 and SCID-5-AMPD-I); a self-report personality functioning score (LPFS-SR); an interview-based personality organisation score (STIPO-R); a self-report personality organisation score (IPO-30) and a self-report personality structure score (OPD-SQ). In a parallel analysis using the default values of the fa.parallel function in the R psych package^37^ we compared the eigenvalues of these scores with those from randomly generated data. The number of factors whose eigenvalues exceeded the corresponding random eigenvalues was then used to guide our subsequent exploratory factor analysis of the same five scores.

### Identifying the central dimensions of PDS

**Fig. 1:**
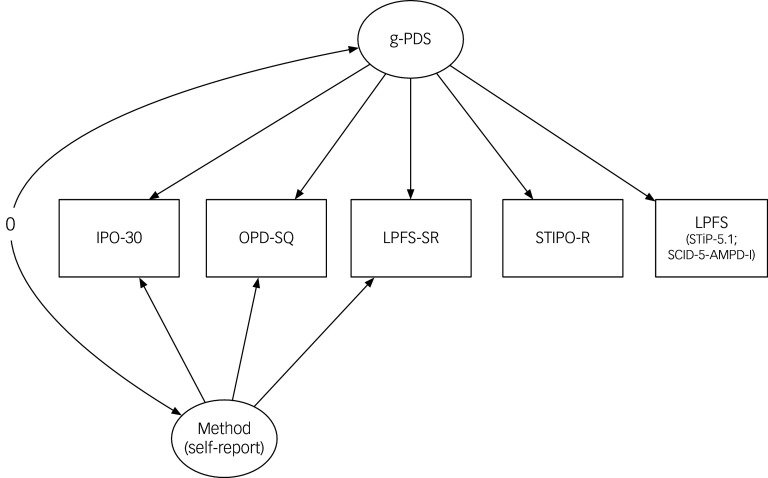
Correlated traits correlated (method – 1) model with a general factor for personality disorder severity (g-PDS), defined by all assessments and an orthogonal self-report factor. IPO-30, Inventory for Personality Organization – 30-item version; OPD-SQ, Operationalized Psychodynamic Diagnostics – Structure Questionnaire; LPFS-SR, Levels of Personality Functioning Scale – Self Report; STIPO-R, Structured Interview for Personality Organization – Revised; STiP-5.1, Semi-structured Interview for Personality Functioning DSM-5; SCID-5-AMPD-I, Structured Clinical Interview for the Alternative DSM-5 Model for Personality Disorders – Module I.

Following the identification of self- and observer-assessment method variance, we constructed a correlated traits correlated (method – 1) model (CTC(M–1)^38^) with a g-PDS factor loading on all dimensions of the above-described assessments indicating PDS (interview and self-report) and an orthogonal factor, which loaded only on self-reported dimensions (see Fig. 1) using the R package lavaan.^39^ By this means, we extracted a g-PDS factor using two different assessment methods and three different conceptualisations of PDS that is most closely aligned with the gold-standard of interview and clinician-based assessment of PDS. The CTC(M–1) model was chosen because it helps separate the effects of different assessment methods by using the interview as a reference method. In such a model, high factor loadings on the interview factor and low factor loadings on the self-report factor indicate PDS dimensions that have high convergence with g-PDS. If a PDS dimension exhibits this pattern both in self- and observer-assessments, then that PDS dimension demonstrates high convergence between assessment methods. Additionally, the model also allows for the investigation of dimensions that capture self-report-specific variance beyond the interview assessment while avoiding overfactorisation issues that can occur in other multitrait-multimethod (MTMM) models.

First, we examined the factor loadings on the g-PDS factor of the CTC(M–1) model to identify the PDS dimensions that capture most of the g-PDS variance. Investigating which self-reported PDS dimensions show greater or equal loadings on the self-report factor than on the g-PDS factor allowed conclusions on diverging variance sources between interview- and self-report assessment. Based on the ranking identified by the g-PDS loadings in the CTC(M–1) model, multiple linear regression models were calculated by sequentially taking manifest scores of the respective PDS dimensions into the model. Using this approach, it was possible to approximate which combination of PDS may suffice to approximate g-PDS most closely.

Second, a latent PDS score was calculated using only the interview-based assessments. Using the factor scores of the interview-based PDS factor as the dependent variable, the Best Items Scale that is Cross-validated, Unit-weighted, Informative and Transparent (BISCUIT) algorithm^40^ was used to calculate bootstrapped correlations with self-reported PDS dimensions. Based on the ranking of the self-reported PDS dimensions with the highest factor loadings identified by the BISCUIT algorithm, multiple linear regression models were calculated by sequentially taking additional dimensions into the model. We interpreted the correlations’ and standardised factor loadings’ effect sizes as follows: small 0.10, medium 0.30 and large 0.50.

Minding the bias of most fit indices in models with high average factor loadings and many parameters^41^ which applies to the CTC(M–1) model used in our study, model fit for the factor analytical models was assessed through the unbiased standardised root mean square residual (uSRMR) using a cut-off value of 0.1 times the average *R* ^2^ of manifest variables.^42^ Multicollinearity among multiple regression models to investigate variance explanation for g-PD was investigated using the vif() function of the car R package^43^ with reported *R* ^2^ values adjusted using *Wherry Formula-1*.

## Results

### Self- and interview-assessment method factors

All assessments of PDS dimensions were normally distributed, except for the IPO-30 domain aggression (skewness: 2.1; kurtosis: 10.3). Correlations of the LPFS interview total score with the total scores of the other assessments ranged from medium associations with the IPO-30 (*r* = 0.47) to strong correlations with the LPFS-SR (*r* = 0.77), STIPO-R (*r* = 0.81) and OPD-SQ (*r* = 0.69) total scores (see Table 2). Based on the total scores of the five assessments, parallel analysis suggested extracting two factors, with the first factor showing, predominantly, loadings of self-report questionnaires (OPD-SQ, LPFS-SR and IPO-30) and the second factor showing loadings of interviews (SCID-5-AMPD-I and STiP-5.1 combined for LPFS and STIPO-R). Following this analysis, we constructed a CTC(M–1) model (Fig. 1), with the g-PDS factor defined by the average scores of all assessments and an orthogonal self-report factor defined by self-report assessments yielding good model fit (uSRMR 0.024, cut-off 0.076). To investigate loadings and variance explanations of g-PDS by all PDS dimensions and subdomains, we additionally constructed a latent CTC(M–1) model with 12 LPFS subdomains (for both self-report and interview, respectively), 6 STIPO-R domains, 5 IPO-30 domains and 8 OPD-SQ domains, again with an orthogonal self-report factor defined by all self-report assessments. This procedure yielded a strong and reliable g-PDS factor (explained common variance 73.0%, ω hierarchical 0.82).

**Table 2:** Correlations between aggregated mean scores of IPO-30, LPFS interviews, LPFS-SR, OPD-SQ and STIPO-R

	LPFS interview	LPFS-SR	STIPO-R	IPO-30
LPFS-SR	0.77			
STIPO-R	0.81	0.66		
IPO-30	0.47	0.66	0.48	
OPD-SQ	0.69	0.80	0.52	0.58

IPO-30, Inventory of Personality Organization – 30 item version; LPFS Interview, semi-structured Interview for Personality Functioning DSM-5 and Structured Clinical Interview for the Alternative DSM-5 Model for Personality Disorders – Module I; LPFS-SR, Level of Personality Functioning – Self Report; OPD-SQ, Operationalized Psychodynamic Diagnosis – Structured Questionnaire; STIPO-R, Structured Interview for Personality Organization – Revised. All correlations show *p*-values <0.01.

### Approximation of g-PDS by interview and self-reported PDS dimensions

**Fig. 2:**
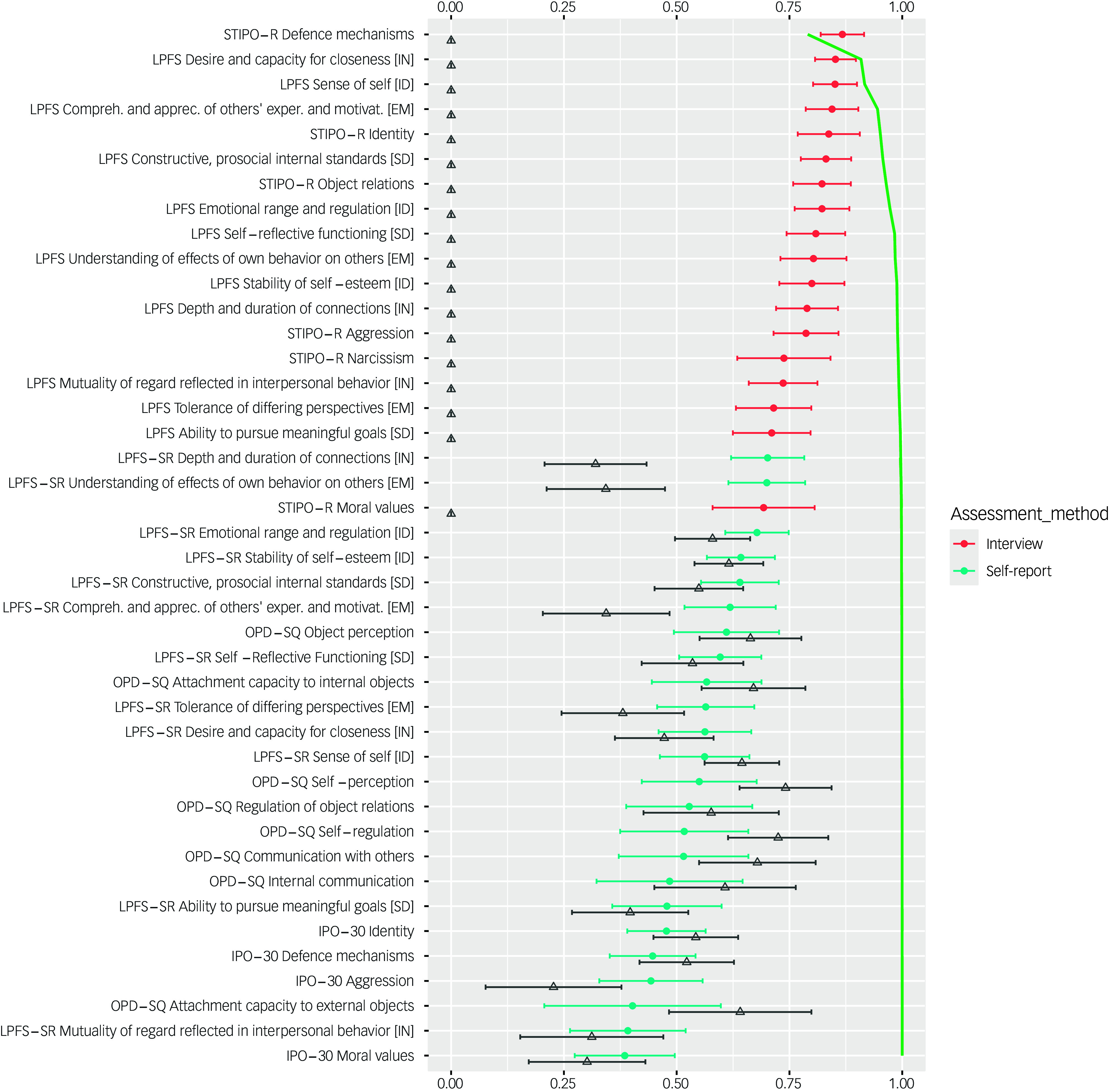
Standardised loadings of self-reported (blue) and interview-assessed (red) personality disorder severity (PDS) dimensions on latent PDS and self-report (triangles) factors of CTC(M–1) model including 95% confidence intervals, adjusted for the missing width inflation factor^33^ *R* ^2^ of sequential multiple linear regressions of manifest PDS subdimension scores predicting latent PDS factor score (green). IPO-30, Inventory of Personality Organization – 30 item version; LPFS Interview, Semi-structured Interview for Personality Functioning DSM-5 and Structured Clinical Interview for the Alternative DSM-5 Model for Personality Disorders – Module I; LPFS-SR, Level of Personality Functioning – Self Report; OPD-SQ, Operationalized Psychodynamic Diagnosis – Structure Questionnaire; STIPO-R, Structured Interview for Personality Organization – Revised; ID, identity; SD, self-direction; EM, empathy; IN, intimacy. Colour online only.

**Fig. 3:**
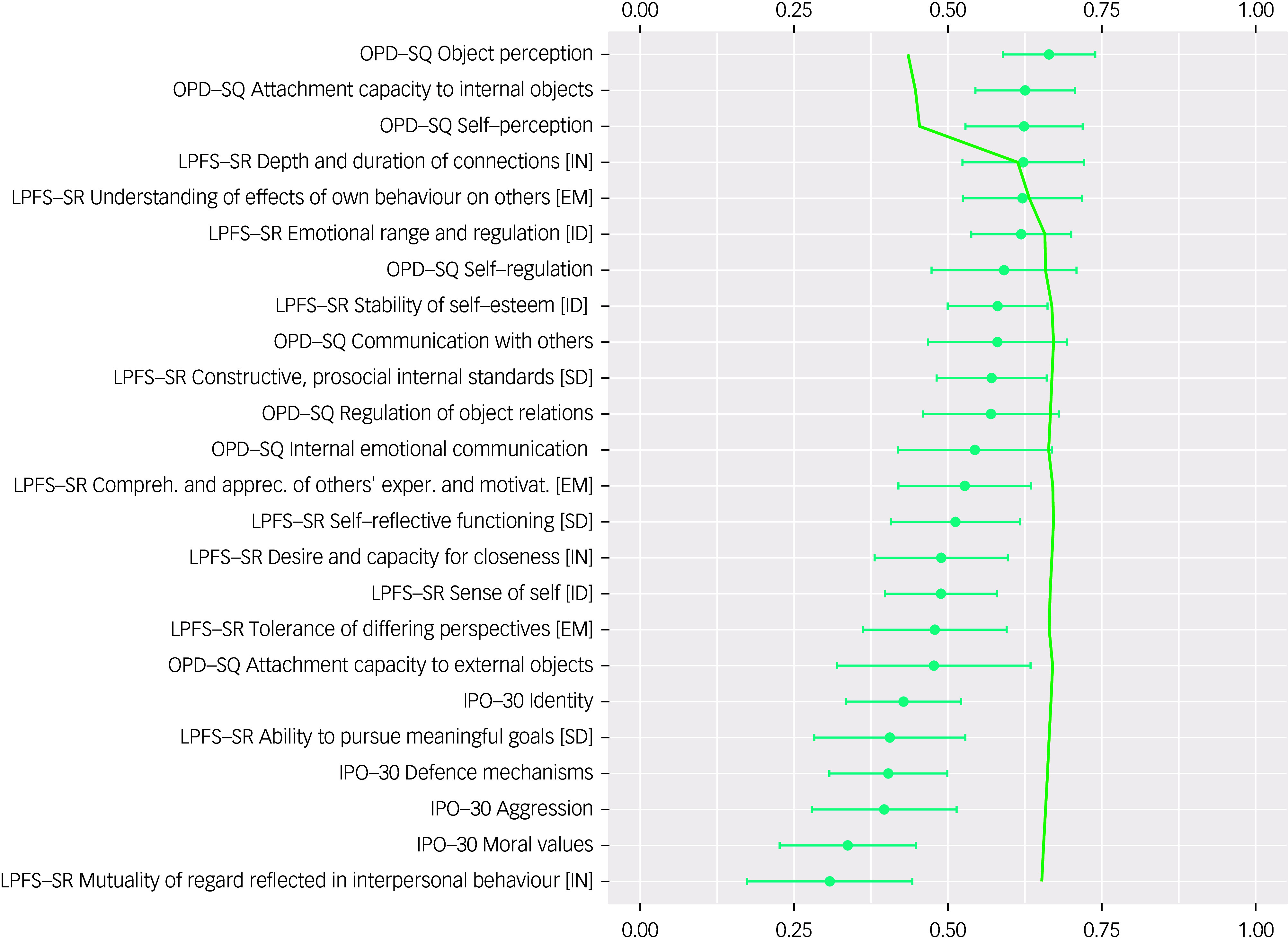
Standardised loadings (blue) and *R* ^2^ (green) of sequential multiple linear regressions of self-reported personality disorder severity (PDS) dimensions predicting latent interview-based PDS factor scores including 95% confidence intervals, adjusted for the missing width inflation.^33^ IPO-30, Inventory for Personality Organization – 30-item version; OPD-SQ, Operationalized Psychodynamic Diagnostics – Structure Questionnaire; LPFS-SR, Level of Personality Functioning Scale – Self Report; ID, identity; SD, self-direction; EM, empathy; IN, intimacy. Colour online only.

Figure 2 shows standardised factor loadings (blue and red circles in Fig. 2) and sequential *R* ^2^ of multiple regression models with the g-PDS factor as a dependent variable and manifest self- and interview-assessed PDS dimensions as independent variables (green line). The highest standardised factor loadings were found for the interview-assessed dimensions of PDS. The four PDS dimensions with highest loadings on g-PDS were the STIPO-R domain *defence mechanisms*, the LPFS intimacy subdomain *desire and capacity for closeness,* the LPFS identity subdomain *sense of self* and the LPFS empathy subdomain *comprehension and appreciation of others’ experiences and motivations*. A combination of either two of these four dimensions explained between 81.8% (*defence mechanisms* and *sense of self*) and 91.3% (*defence mechanisms* and *desire and capacity for closeness*). Regarding self-reports, the OPD-SQ dimensions *object perception, attachment capacity to internal objects* and self-perceptions as well as the LPFS-SR dimension *depth and duration of connections* predicted 61.3% of the variance of the latent interview-based g-PDS score, with all investigated self-reported dimensions together adding up to 65.2% variance explanation (adjusted *R* ^2^). Detailed results can be found in Fig. 3. All multiple regression models with less than 27 predictors had an average variance inflation factor of less than 5, indicating that multicollinearity affected all regression models with 27 predictors or above (see Supplementary material).

### Dimensions of PDS with substantial self-report assessment method variance

All self-reported PDS dimensions showed at least medium method variance, i.e. factor loadings >0.30 on the self-report factor, except for the LPFS-SR empathy subdomains *understanding of effects of own behaviour on others* and *comprehension and appreciation of others’ experiences and motivations,* the LPFS-SR intimacy subdomain *depth and duration of connections* and the IPO-30 domains *moral values* and *aggression*, showing standardised factor loadings <0.30 on the self-report factor. All eight OPD-SQ domains as well as several LPFS-SR subdomains of the identity and self-direction domains and the IPO-30 domains *identity* and *primitive defences* showed large method variance with standardised factor loadings >0.50 on the self-report factor (see grey triangles in Fig. 2). The IPO-30 domain *reality testing* showed low correlations with g-PDS (*r* = 0.29) and was therefore omitted.

## Discussion

The aim of this study was to determine the empirically sound core dimensions of PDS in self-reported and interview-based assessments as well as to identify dimensions with significant self-report variance. These core dimensions can provide a basis for future studies on short PDS assessments for time-limited settings and on etiological investigations on the core aspects of personality disorder. To this end, we assessed a mixed clinical and non-clinical sample with three semi-structured interviews and three self-reports for personality functioning, personality organisation and personality structure.

### Essential dimensions of a latent g-PDS factor

Within interview-assessed PDS, the LPFS intimacy subdomain *capacity and desire for closeness,* and within self-reported PDS, the LPFS intimacy subdomain *depth and duration of connections* showed a high variance explanation of g-PDS. Previous investigations using the Social Cognition and Object Relations Scale (SCORS)^44^ which influenced the definition of the DSM-5 LPFS intimacy domain^5^ already found that the dimension *capacity for emotional investment in relationships* is highly indicative for PD. These findings align with object relations theory’s core assertion that early adversities in the interplay between an individual’s inherent temperament and environmental interpersonal resources may shape maladaptive self- and other representations as well as over-representations of relationships predominated by negative effects, which is especially detrimental for (future) intimate relationships.^45^ More recent investigations of other authors even postulate that the term ‘personality disorder’ should be replaced by interpersonal disorders^46,47^ reflecting the centrality of the interpersonal etiology and symptomatology in most personality disorders, which is further underlined by our findings of the high convergence of the OPD-SQ dimensions *object perception* and *attachment capacity to internal objects* with g-PDS.

In addition, our results show that *defence mechanisms* were another highly predictive dimension to approximate g-PDS. These results are consistent with previous findings^19,26^ and align with Kernberg’s model of personality organisation, according to which defence mechanisms (especially primitive defences) are a central indicator of PDS.^30^ In general, *defence mechanisms* can be understood as unconscious and highly automated psychological processes to deal with internal conflicts and stressful events.^48^ These can have more mature forms, such as humour or intellectualisation, or immature forms, such as projection or denial.^48^ Additionally, the high discrepancy between g-PDS loadings of interview-assessed STIPO-R *defence mechanisms* and the self-rated IPO-30 domain *primitive defence* in company with the significant self-report method variance in our results suggests that to reliably capture defence mechanisms, an assessment by an experienced clinician may be needed (see the Supplementary material for an illustrative case example).

Our results also reveal that another PDS dimension capturing large amounts of g-PDS variance is the interview-assessed LPFS subdomain *sense of self*. This result corresponds to existing evidence on associations of the identity domain and PDS.^49^ Interestingly, the two remaining interview-assessed LPFS identity subdomains (*self-esteem* and *emotional range and regulation)* and the STIPO-R identity domain also showed a high predictive value (*λ* > 0.65), but explain little additional variance if added to the LPFS subdomain *sense of self* in a sequential regression model, corroborating the high convergence of this construct within the frameworks of personality functioning and personality organisation.^26^ *Sense of self* refers to a continuous, differentiated, coherent experience of an authentic, vital subject with boundaries to others. It is important to note that all LPFS identity subdomains showed moderate to strong self-report variance, suggesting that these PDS dimensions should be assessed using a clinician-based assessment. However, there is also evidence that, specifically, personality disorder aspects related to identity, affective instability and feelings of emptiness may even be assessed more reliably using self-report.^50^ Notably, despite moderate self-report variance, the LPFS-SR dimension *emotional range and regulation* strongly predicted interview-assessed PDS, highlighting its reliability. Future longitudinal research therefore needs to investigate if the identity domain may best be captured through a combination of both self- and observer-based methods.^51^

Regarding findings of the high g-PDS convergence of the interview-assessed empathy subdomain *comprehension and appreciation of others’ experiences and motivations,* and the self-reported subdomain *understanding of effects of own behaviour on others*, our results are consistent with recent findings that impairments in empathy are a marker of general personality pathology.^52^ These LPFS subdimensions trace back to the incorporation of the mentalisation concept within the LPFS empathy domain.^5^ Mentalisation concerns the ability to perceive and interpret one’s own and others’ internal mental states, encompassing feelings, thoughts and motives, which is highly associated with personality disorder and psychopathology in general.^53^ Notably, empathy deficits may result from identity diffusion, where emotional contagion hinders genuine understanding^54^ emphasising the interplay between the subdomains.

It is important to note that personality functioning as defined by the DSM-5 LPFS does not contain self- or other injurious behaviour, which PDS as defined by the ICD-11 does. However, the dimension of self- and other-directed aggression is a central part of Kernberg’s model and is operationalised by the STIPO-R and IPO-30. In addition, our analyses point to the direction that aggression, both self-reported and interview-assessed, is an important indicator of g-PDS. Another distinction is ICD-11’s inclusion of psychosocial functioning, though the STIPO-R identity domain, which strongly loads on g-PDS, covers investment in work or other activities as a proxy for participation.

### Implications for clinical routine

Our findings suggest that impairments in identity and intimacy are highly indicative to approximate PDS. Defence mechanisms are the most indicative dimension, but are not part of international classification systems, It may therefore be worthwhile to get training in the assessment of defence mechanisms, e.g. by learning the administration of the STIPO-R. Notably, defences can also be seen as highly automatised processes of emotion regulation, relevant to various therapeutic approaches and a central diagnostic factor. More specifically, according to Kernberg’s model, defence mechanisms distinguish between high (primitive splitting-based defence mechanisms) and low personality dysfunction (mature defences). Future research needs to investigate the nomological net of defence mechanisms within other contemporary treatment concepts such as mentalisation or schema therapy.

In addition, the PDS dimensions identity, emotion regulation, empathy, desire for closeness as well as defence mechanisms were found to be highly predictive for general psychopathology.^55,56^ Routine assessment of the centrally indicative PDS dimensions found in this study may therefore be beneficial in case conceptualisations for all kinds of psychopathology.

As an example, interview questions to assess the degree to which the individual experiences itself as unique with clear boundaries between self and others (DSM-5 LPFS identity subdomain *sense of self*) could be: ‘How would you describe yourself as a person?’or ‘What kind of person are you?’ (STiP-5.1^14^). To assess the need and ability for emotional closeness with others (DSM-5 LPFS intimacy subdomain *desire and capacity for closeness*) questions such as ‘Is it easy for you to open up in relationships?’ may be helpful (SCID-5-AMPD-I^13^). To assess the degree to which the individual understands and appreciates others’ experiences and motivations (DSM-5 LPFS empathy subdomain), a question such as ‘Do you usually know what makes other people tick and why they do the things they do?’ (SCID-5-AMPD-I^13^) may be helpful. To assess conscious, affective, cognitive and behavioural correlates of primitive defence mechanisms^48^ the question ‘Have people pointed out that you tend to blame others or circumstances, for things that happen to you, or that you have difficulty accepting responsibility for your actions?’ (STIPO-R^10^) could be helpful. Please refer to the respective SCID-5-AMPD-I, STiP-5.1 or STIPO-R manuals for information on rating anchors to these questions. In addition, we provide a case vignette in the Supplementary material exemplifying the above outlined assessment procedure. Further training materials concerning the above-mentioned interviews can be requested from the first and last authors, A.K. and S.H.-S., via the former.

### Limitations

Some limitations should be noted. First, comparing the predictive power of PDS dimensions assessed in a chronological structured interview requires caution, as implicit knowledge from earlier domains (identity, self-direction and empathy) may influence the rating of intimacy, which is assessed last. Second, the variance explanation found for the interview-assessment of the g-PDS in the multiple regression is inflated, as we predict a latent, interview-based score with the dimensions of these interviews. However, both interview-assessed and self-reported LPFS intimacy subdomains exhibited highest correlations with g-PDS, both with interview and self-reports modeled in one model and separately, suggesting that this finding is no artifact of an assessment or modeling method. Third, the construction of the model lacked an interview for personality structure according to the OPD. This probably led to a specific variance of the OPD conceptualisation of personality functioning to be allocated in the self-report factor and therefore not represented in g-PDS. Despite this methodological problem, two of the OPD domains (*object perception* and *attachment capacity to internal objects*) showed strong loadings on g-PDS. Further PDS dimensions that showed moderate to high self-report variance (e.g. LPFS-SR *self-esteem* and *sense of self*) may therefore also reflect aspects of PDS where self-reports may yield incremental and unique additional variance.^57^

### Implications of the study

Profound personality disorder diagnosis is time-consuming and challenging. Therefore, the present study determined the essential dimensions for the clinical approximation of PDS using three semi-structured interviews and three self-reports of personality functioning, personality organisation and personality structure. This multi-method procedure ensured validity, reflecting a growing consensus that personality pathology should be assessed by a combination of self-reports and clinical interviews.^51^ As a result, we identified the self-reported LPFS dimensions, *depth and duration of connections* and *attachment capacity to internal objects*, as well as the OPD-SQ domains *self-perception* and *object perception*, to show highest convergence with g-PDS. The four interview-assessed dimensions *defence mechanisms* (STIPO-R), *desire and capacity for closeness* (LPFS domain intimacy), *sense of self* (LPFS domain identity) and *comprehension and appreciation of others’ experiences and motivations* (LPFS domain empathy) were revealed to be highly indicative of g-PDS. Taken together, we recommend assessing at least the latter three LPFS dimensions, which do not require extensive training^58^ when time is limited. However, the evaluation of defence mechanisms may also be fruitful. Future research should therefore investigate how and if knowledge of psychodynamic concepts (e.g. defence mechanisms) could improve clinical utility when assessing PDS, and how a multi-method short PDS assessment focused on the dimensions identified in this study performs psychometrically and clinically.

## Supporting information

Kerber et al. supplementary material 1 Kerber et al. supplementary material

Kerber et al. supplementary material 2 Kerber et al. supplementary material

Kerber et al. supplementary material 3 Kerber et al. supplementary material

## Data Availability

The data that support the findings of this study are openly available in the Open Science Framework at https://osf.io/bhq94/

## Supplementary material

The supplementary material is available online at https://doi.org/10.1192/bjp.2025.10347
