# Supplementary material for: Determining essential dimensions for the clinical approximation of personality disorder severity: multi-method study
Source: Br J Psychiatry. 2025 Sep 24;228(1):46–54. doi: 10.1192/bjp.2025.10347 (PMC12722010; doi:10.1192/bjp.2025.10347)
Supplement: Kerber et al. supplementary material 1 — Kerber et al. supplementary material [file S0007125025103474sup001.docx]

**Supplementary Material:** Case example

**This case example is a demonstration of the use of questions taken from Module I of the SCID-5-AMPD Interview** [**(Bender et al. 2018)**](https://www.zotero.org/google-docs/?DbM7It)**, the STip 5.1 semi-structured interview (Hutsebaut et al., 2017) , and the STIPO-R (Clarkin et al., 2016) interview.**

**___________________________________________________________________________**

**Interview with Ian, a 32-Year-Old Artist, who is currently going through a divorce, which has left him struggling with severe symptoms of depression. He seeks treatment to address his intense feelings of emptiness, self-doubt, and hopelessness.**

___________________________________________________________________________

Ian sat across from the interviewer, his posture tense, arms wrapped around himself protectively. His gaze alternated between defiance and detachment, as if he were bracing for an uncomfortable discussion yet unwilling to fully engage. The past months had been marked by emotional upheaval, and though he tried to present a composed exterior, a deep uncertainty lay beneath the surface.

**Sense of Self**

**Interviewer: How would you describe yourself as a person? What kind of a person are you?** (*STiP 5.1 Question 1.1.1*)

**Ian:** I guess I’m passionate. When I’m inspired, I throw myself into my art completely. People say I’m intense.
**Interviewer:** What does being “intense” mean to you?
**Ian:** I feel things deeply—more than most people, I think. But sometimes… I don’t really know who I am. When I’m not creating or when I’m alone, I feel… sort of empty.

**Interviewer: Do you tend to feel empty much of the time?** (*SCID-5-AMPD-I, p.12, DSM-5 LPFS identity subdomain sense of self with boundaries to others*)

**Ian:** Yeah. When things are good, I’m fine. But when something throws me off—like this divorce—it’s like there’s nothing left inside.

**Interviewer: Do you sometimes completely lose the sense of who you are when interacting with others?** (*SCID-5-AMPD-I, p.12, DSM-5 LPFS identity subdomain sense of self with boundaries to others*)

**Ian:** I wouldn’t say “lose” exactly… but I change. Depending on who I’m with, I can be different. It’s not fake—I just don’t know what’s really me.

Ian’s response reflects **a severely impaired sense of self (LPFS: Identity, Level 3 - Severe)**. He relies heavily on external validation—his artistic output and relationships—to maintain a stable self-concept. When these are disrupted, he experiences profound emptiness and self-doubt. His difficulty in defining himself independently of his work and relationships suggests a **fragile and fragmented identity**, characteristic of severe personality dysfunction.

His experience of shifting self-perception based on external factors reinforces **his unstable identity structure**. The **severe sense of emptiness and fluid self-concept** he describes suggest that his identity is highly reactive to relational and situational contexts, making it difficult for him to maintain a consistent inner sense of self. He experiences chronic feelings of emptiness and struggles to maintain a stable identity, often adapting to those around him. Without external validation—through relationships or creative expression—he feels nonexistent. This extreme instability in self-concept significantly impairs his ability to navigate life independently.

**Comprehension and appreciation of others’ experiences and motivations**

**Interviewer: Do you usually know what makes other people tick and why they do the things they do?** (*SCID-5-AMPD-I Screener Question, p.24*)

**Ian:** Yeah, I’m pretty good at reading people. I can tell what they want or expect—especially when it comes to me.
**Interviewer:** And do you think your understanding of others comes more from genuine interest or as a way to protect yourself?
**Ian:** [pauses] Maybe both. I like to know where I stand with people. If I can anticipate what they’ll do, I won’t get caught off guard.

**Interviewer: Are you interested in what other people say or do mostly so you can take care of yourself and your own interests?** (*SCID-5-AMPD-I, p.25, DSM-5 LPFS empathy subdomain comprehension and appreciation of others’ experiences and motivations*)

**Ian:** That sounds selfish, but… yeah, I guess. If I don’t watch out for myself, who will?

His response further reinforces **his self-focused approach to relationships**. While he does not entirely lack interest in others, his engagement is primarily shaped by his own needs and insecurities, rather than by a balanced perspective that considers both his and others’ experiences.

Ian’s response suggests **moderate impairment in empathy (LPFS: Comprehension and Appreciation of Others, Level 2 - Moderate)**. While he has an intuitive sense of people’s motivations, it is not primarily driven by curiosity or emotional attunement, but rather by **self-preservation**. His interpersonal awareness is largely defensive, aiming to **preempt rejection or betrayal** rather than foster genuine emotional connection.

**Desire and capacity for closeness**

**Interviewer: Is it easy for you to open up in relationships?** (*SCID-5-AMPD-I Screener Question, p.34*)

**Ian:** Not really. I mean, I can talk about stuff, but deep down? I don’t know if anyone really gets me.

**Interviewer: Is it important for you to try to get close to people so that you can feel appreciated or admired?** (*SCID-5-AMPD-I, p.34, DSM-5 LPFS intimacy subdomain desire and capacity for closeness*)

**Ian:** Yeah… I guess that’s part of it. It feels good when someone values me. But sometimes, when I get too close, I push them away.

Ian’s **moderate impairment in intimacy (LPFS: Intimacy, Level 2 - Moderate)** is characterized by **a push-pull dynamic**—while he seeks admiration and validation from others, true vulnerability is difficult for him. His **fear of rejection or engulfment leads to distancing behaviors**, which in turn perpetuate his feelings of isolation.

**Defense mechanisms**

**Interviewer: Have people pointed out that you tend to blame others or circumstances for things that happen to you, or that you have difficulty accepting responsibility for your actions?** (*STIPO-R; Primitive Defense Mechanism of 'externalization', Question 34*)

**Ian:** People love to say that. My ex, for example—she claimed I was always “making myself the victim.” But what was I supposed to do? She was going to leave me. I just got ahead of it. And also concerning my work, I mean, things happen for a reason. One of the main reasons my career isn’t working out to date, it’s because people don’t appreciate real art anymore.

This response demonstrates **primitive defense mechanisms, particularly externalization and projective identification**. Ian shifts responsibility for his difficulties onto external factors, minimizing his own role in conflicts (**externalization**). Additionally, his **preemptive breakup** to avoid being abandoned suggests **projective identification**—by ending the relationship first, he confirms his own fear of rejection, maintaining a sense of control while recreating the very outcome he dreads. By acting out his anxieties in this way, he unwittingly shapes his relationships in a self-fulfilling cycle of loss and rejection. ca
