# Supplementary material for: Determining essential dimensions for the clinical approximation of personality disorder severity: multi-method study
Source: Br J Psychiatry. 2025 Sep 24;228(1):46–54. doi: 10.1192/bjp.2025.10347 (PMC12722010; doi:10.1192/bjp.2025.10347)
Supplement: Kerber et al. supplementary material 2 — Kerber et al. supplementary material [file S0007125025103474sup002.docx]

**Supplementary Table 1.** *Pairs of Assessments*

|  | IPO-30 | LPFS interview | LPFS-SR | OPD-SQ | STIPO-R |
| --- | --- | --- | --- | --- | --- |
| IPO-30 | 465 | 235 | 330 | 176 | 281 |
| LPFS interview | 235 | 291 | 107 | 162 | 57 |
| LPFS-SR | 330 | 107 | 334 | 130 | 281 |
| OPD-SQ | 176 | 162 | 130 | 186 | 78 |
| STIPO-R | 281 | 57 | 281 | 78 | 295 |
| *Notes.* IPO–30 = Inventory of Personality Organization – 30 item version. LPFS interview = Semi Structured Interview for Personality Functioning DSM–5 and Structured Clinical Interview for the Alternative DSM–5 Model for Personality Disorders – Module I. LPFS–SR = Level of Personality Functioning – Self Report. OPD-SQ = Operationalized Psychodynamic Diagnosis – Structured Questionnaire. STIPO–R = Structured Interview for Personality Organization – Revised. | | | | | |
