# Supplementary material for: Determining essential dimensions for the clinical approximation of personality disorder severity: multi-method study
Source: Br J Psychiatry. 2025 Sep 24;228(1):46–54. doi: 10.1192/bjp.2025.10347 (PMC12722010; doi:10.1192/bjp.2025.10347)
Supplement: Kerber et al. supplementary material 3 — Kerber et al. supplementary material [file S0007125025103474sup003.docx]

**Supplementary Table 2A.** Scale parameters including fraction of missing information, CTCM-1 model loadings and variance inflation factor

|  | g_PD | self-report factor | gPD.ci.lower | gPD.ci.upper | Meth.ci.lower | Meth.ci.upper | FMI | Assessment_method | rsqu | VIF |
| --- | --- | --- | --- | --- | --- | --- | --- | --- | --- | --- |
| STIPO-R Defense mechanisms | 0.867 | 0.000 | 0.819 | 0.915 | 0.000 | 0.000 | 0.411 | interview | 0.791 | NA |
| LPFS Desire and capacity for closeness [IN] | 0.852 | 0.000 | 0.807 | 0.897 | 0.000 | 0.000 | 0.373 | interview | 0.909 | 2.230 |
| LPFS Sense of self [ID] | 0.851 | 0.000 | 0.802 | 0.900 | 0.000 | 0.000 | 0.357 | interview | 0.917 | 2.423 |
| LPFS Comprehension and appreciation of others’ experiences and motivations [EM] | 0.844 | 0.000 | 0.786 | 0.903 | 0.000 | 0.000 | 0.370 | interview | 0.945 | 2.612 |
| STIPO-R Identity | 0.837 | 0.000 | 0.768 | 0.906 | 0.000 | 0.000 | 0.431 | interview | 0.952 | 3.179 |
| LPFS Constructive, prosocial internal standards of behavior [SD] | 0.831 | 0.000 | 0.775 | 0.887 | 0.000 | 0.000 | 0.420 | interview | 0.957 | 3.221 |
| STIPO-R Object relations | 0.822 | 0.000 | 0.758 | 0.886 | 0.000 | 0.000 | 0.453 | interview | 0.965 | 3.425 |
| LPFS Emotional range and regulation [ID] | 0.822 | 0.000 | 0.762 | 0.883 | 0.000 | 0.000 | 0.392 | interview | 0.973 | 3.351 |
| LPFS Self-Reflective Functioning [SD] | 0.809 | 0.000 | 0.744 | 0.873 | 0.000 | 0.000 | 0.391 | interview | 0.983 | 3.252 |
| LPFS Understanding of effects of own behavior on others [EM] | 0.803 | 0.000 | 0.730 | 0.877 | 0.000 | 0.000 | 0.404 | interview | 0.984 | 3.368 |
| LPFS Self-esteem [ID] | 0.800 | 0.000 | 0.728 | 0.872 | 0.000 | 0.000 | 0.408 | interview | 0.988 | 3.522 |
| LPFS Depth and duration of connections [IN] | 0.789 | 0.000 | 0.720 | 0.858 | 0.000 | 0.000 | 0.412 | interview | 0.989 | 3.607 |
| STIPO-R Aggression | 0.787 | 0.000 | 0.715 | 0.859 | 0.000 | 0.000 | 0.404 | interview | 0.990 | 3.569 |
| STIPO-R Narcissism | 0.738 | 0.000 | 0.634 | 0.841 | 0.000 | 0.000 | 0.468 | interview | 0.991 | 3.637 |
| LPFS Mutuality of regard reflected in interpersonal behavior [IN] | 0.736 | 0.000 | 0.660 | 0.812 | 0.000 | 0.000 | 0.433 | interview | 0.992 | 3.570 |
| LPFS Tolerance of differing perspectives [EM] | 0.715 | 0.000 | 0.631 | 0.798 | 0.000 | 0.000 | 0.432 | interview | 0.994 | 3.632 |
| LPFS Ability to pursue meaningful goals [SD] | 0.711 | 0.000 | 0.625 | 0.797 | 0.000 | 0.000 | 0.440 | interview | 0.996 | 3.639 |
| LPFS-SR Depth and duration of connections [IN] | 0.702 | 0.320 | 0.620 | 0.783 | 0.207 | 0.433 | 0.377 | sel-freport | 0.996 | 3.680 |
| LPFS-SR Understanding of effects of own behavior on others [EM] | 0.700 | 0.343 | 0.615 | 0.785 | 0.212 | 0.474 | 0.367 | self-report | 0.998 | 3.728 |
| STIPO-R Moral values | 0.693 | 0.000 | 0.580 | 0.806 | 0.000 | 0.000 | 0.464 | interview | 0.998 | 3.816 |
| LPFS-SR Emotional range and regulation [ID] | 0.678 | 0.580 | 0.608 | 0.748 | 0.496 | 0.663 | 0.341 | self-report | 0.998 | 3.911 |
| LPFS-SR Self-esteem [ID] | 0.642 | 0.616 | 0.567 | 0.718 | 0.539 | 0.692 | 0.344 | sel-freport | 0.999 | 4.184 |
| LPFS-SR Constructive, prosocial internal standards of behavior [SD] | 0.640 | 0.549 | 0.554 | 0.726 | 0.451 | 0.647 | 0.428 | sel-freport | 0.999 | 4.393 |
| LPFS-SR Comprehension and appreciation of others’ experiences and motivations [EM] | 0.618 | 0.344 | 0.517 | 0.720 | 0.203 | 0.484 | 0.405 | self-report | 0.999 | 4.527 |
| OPD-SQ Object perception | 0.610 | 0.664 | 0.494 | 0.727 | 0.551 | 0.776 | 0.484 | interview | 0.999 | 4.691 |
| LPFS-SR Self-Reflective Functioning [SD] | 0.597 | 0.535 | 0.505 | 0.688 | 0.422 | 0.648 | 0.358 | sel-freport | 0.999 | 4.726 |
| OPD-SQ Attachment capacity to internal objects | 0.566 | 0.670 | 0.445 | 0.688 | 0.555 | 0.785 | 0.509 | interview | 0.999 | 5.010 |
| LPFS-SR Tolerance of differing perspectives [EM] | 0.564 | 0.381 | 0.457 | 0.672 | 0.245 | 0.516 | 0.429 | self-report | 1.000 | 5.045 |
| LPFS-SR Desire and capacity for closeness [IN] | 0.563 | 0.472 | 0.460 | 0.665 | 0.363 | 0.582 | 0.397 | self-report | 1.000 | 5.561 |
| LPFS-SR Sense of self [ID] | 0.562 | 0.645 | 0.463 | 0.661 | 0.562 | 0.727 | 0.358 | self-report | 1.000 | 6.012 |
| OPD-SQ Self-Perception | 0.550 | 0.741 | 0.423 | 0.677 | 0.639 | 0.843 | 0.477 | interview | 1.000 | 6.351 |
| OPD-SQ Regulation of object-relations | 0.528 | 0.576 | 0.388 | 0.668 | 0.426 | 0.726 | 0.531 | interview | 1.000 | 6.792 |
| OPD-SQ Self-regulation | 0.517 | 0.725 | 0.375 | 0.659 | 0.614 | 0.836 | 0.484 | interview | 1.000 | 7.736 |
| OPD-SQ Communication with others | 0.515 | 0.679 | 0.371 | 0.659 | 0.550 | 0.808 | 0.500 | interview | 1.000 | 7.991 |
| OPD-SQ Internal communication | 0.484 | 0.607 | 0.322 | 0.646 | 0.450 | 0.764 | 0.558 | interview | 1.000 | 8.913 |
| LPFS-SR Ability to pursue meaningful goals [SD] | 0.478 | 0.397 | 0.357 | 0.600 | 0.268 | 0.526 | 0.408 | sel-freport | 1.000 | 9.498 |
| IPO-30 Identity | 0.477 | 0.542 | 0.390 | 0.564 | 0.448 | 0.636 | 0.222 | sel-freport | 1.000 | 10.281 |
| IPO-30 Defenses Mechanisms | 0.447 | 0.522 | 0.352 | 0.542 | 0.418 | 0.627 | 0.232 | self-report | 1.000 | 11.066 |
| IPO-30 Aggression | 0.443 | 0.227 | 0.328 | 0.558 | 0.076 | 0.378 | 0.259 | self-report | 1.000 | 11.493 |
| OPD-SQ Internal Attachment Representations capacity to internal objects | 0.402 | 0.641 | 0.206 | 0.598 | 0.483 | 0.799 | 0.541 | interview | 1.000 | 11.974 |
| LPFS-SR Mutuality of regard reflected in interpersonal behavior [IN] | 0.392 | 0.312 | 0.264 | 0.520 | 0.153 | 0.470 | 0.439 | self-report | 1.000 | 12.596 |
| IPO-30 Moral values | 0.385 | 0.301 | 0.274 | 0.496 | 0.172 | 0.431 | 0.238 | self-report | 1.000 | 13.241 |

**Supplementary Table 2B.** Self-reports scales predicting interview score, Scale parameters including fraction of missing information, CTCM-1 model loadings and variance inflation factor

| scales | correlations | confint | FMI | ci.lower | ci.upper | rsqu | VIF | Assessment_method |
| --- | --- | --- | --- | --- | --- | --- | --- | --- |
| OPD-SQ Object perception | 0.662 | 0.079 | 0.222 | 0.584 | 0.741 | 0.435 | NA | self-report |
| LPFS-SR Depth and duration of connections [IN] | 0.625 | 0.078 | 0.259 | 0.547 | 0.703 | 0.591 | 1.450 | self-report |
| OPD-SQ Attachment capacity to internal objects | 0.625 | 0.079 | 0.238 | 0.545 | 0.704 | 0.588 | 2.121 | self-report |
| OPD-SQ Self-Perception | 0.623 | 0.122 | 0.477 | 0.501 | 0.745 | 0.613 | 2.408 | self-report |
| LPFS-SR Understanding of effects of own behavior on others [EM] | 0.623 | 0.105 | 0.440 | 0.517 | 0.728 | 0.632 | 2.467 | self-report |
| LPFS-SR Emotional range and regulation [ID] | 0.619 | 0.080 | 0.357 | 0.539 | 0.699 | 0.658 | 2.701 | self-report |
| OPD-SQ Self-regulation | 0.591 | 0.120 | 0.370 | 0.471 | 0.710 | 0.658 | 2.954 | self-report |
| OPD-SQ Communication with others | 0.581 | 0.105 | 0.373 | 0.476 | 0.686 | 0.663 | 2.931 | self-report |
| LPFS-SR Self-esteem [ID] | 0.581 | 0.087 | 0.433 | 0.494 | 0.669 | 0.672 | 3.049 | self-report |
| LPFS-SR Constructive, prosocial internal standards [SD] | 0.572 | 0.089 | 0.432 | 0.483 | 0.661 | 0.669 | 3.122 | self-report |
| OPD-SQ Regulation of object-relation | 0.569 | 0.110 | 0.392 | 0.458 | 0.679 | 0.666 | 3.147 | self-report |
| OPD-SQ Internal communication | 0.542 | 0.131 | 0.468 | 0.411 | 0.673 | 0.664 | 3.097 | self-report |
| LPFS-SR Comprehension and appreciation of others' experiences and motivations[EM] | 0.531 | 0.112 | 0.404 | 0.419 | 0.643 | 0.670 | 3.143 | self-report |
| LPFS-SR Self-Reflective Functioning [SD] | 0.513 | 0.107 | 0.404 | 0.407 | 0.620 | 0.671 | 3.200 | self-report |
| LPFS-SR Desire and capacity for closeness [IN] | 0.491 | 0.107 | 0.391 | 0.384 | 0.598 | 0.669 | 3.218 | self-report |
| LPFS-SR Sense of self [ID] | 0.489 | 0.092 | 0.232 | 0.396 | 0.581 | 0.666 | 3.307 | self-report |
| LPFS-SR Tolerance of differing perspectives [EM] | 0.478 | 0.115 | 0.412 | 0.363 | 0.593 | 0.665 | 3.330 | self-report |
| OPD-SQ Attachment capacity to external objects | 0.475 | 0.156 | 0.453 | 0.319 | 0.631 | 0.670 | 3.375 | self-report |
| IPO-30 Identity | 0.426 | 0.098 | 0.431 | 0.328 | 0.524 | 0.667 | 3.336 | self-report |
| LPFS-SR Ability to pursue meaningful goals [SD] | 0.404 | 0.123 | 0.464 | 0.282 | 0.527 | 0.664 | 3.299 | self-report |
| IPO-30 Defense mechanisms | 0.402 | 0.103 | 0.420 | 0.299 | 0.505 | 0.662 | 3.309 | self-report |
| IPO-30 Aggression | 0.397 | 0.117 | 0.408 | 0.280 | 0.514 | 0.659 | 3.291 | self-report |
| IPO-30 Moral values | 0.337 | 0.108 | 0.411 | 0.229 | 0.445 | 0.655 | 3.254 | self-report |
| LPFS-SR Mutuality of regard reflected in interpersonal behavior [IN] | 0.308 | 0.141 | 0.484 | 0.168 | 0.449 | 0.652 | 3.227 | self-report |
